# Phytochemical Profiling and Anti-Obesogenic Potential of *Scrophularia aestivalis* Griseb. (Scrophulariaceae)

**DOI:** 10.3390/molecules30214202

**Published:** 2025-10-27

**Authors:** Konstantina Priboyska, Monika N. Todorova, Vanya I. Gerasimova, Martina S. Savova, Slaveya Krustanova, Zhanina Petkova, Stoyan Stoyanov, Milena P. Popova, Milen I. Georgiev, Kalina Alipieva

**Affiliations:** 1 Institute of Organic Chemistry with Centre of Phytochemistry, Bulgarian Academy of Sciences, 1113 Sofia, Bulgaria; priboyskakonstantina@gmail.com (K.P.); vanya.gerasimova@orgchm.bas.bg (V.I.G.); slaveya.krustanova@orgchm.bas.bg (S.K.); zhanina.petkova@orgchm.bas.bg (Z.P.); milena.popova@orgchm.bas.bg (M.P.P.); 2 Laboratory of Metabolomics, Institute of Microbiology, Bulgarian Academy of Sciences, 4000 Plovdiv, Bulgaria; mntodorova@yahoo.com (M.N.T.); m.sav@abv.bg (M.S.S.); 3 Centre of Competence “Sustainable Utilization of Bio-Resources and Waste of Medicinal and Aromatic Plants for Innovative Bioactive Products” (BIORESOURCES BG), 1113 Sofia, Bulgaria; 4 Center of Plant Systems Biology and Biotechnology, 4000 Plovdiv, Bulgaria; 5 Institute of Biodiversity and Ecosystem Research, Bulgarian Academy of Sciences, 1113 Sofia, Bulgaria; tjankata@abv.bg

**Keywords:** *Scrophularia aestivalis* Griseb., *cis*-harpagoside, *trans*-harpagoside, UPLC-HRMS/MS, HPLC method, *Caenorhabditis elegans*, obesity, mitochondrial function

## Abstract

*Scrophularia aestivalis* Griseb. is a Balkan endemic species whose phytochemical composition and medicinal properties have not been previously investigated. The therapeutic potential of *Scrophularia* species has attracted considerable attention, resulting in extensive studies on their chemical and pharmacological properties, with over 200 secondary metabolites identified to date. The present study aimed to explore the phytochemical composition of Bulgarian-origin *S. aestivalis*, including isolation and characterization of individual secondary metabolites. From methanol extract of the plant’s aerial parts, aucubin, harpagide, 8-*O*-acetylharpagide, *cis*- and *trans*-harpagoside, 6-*O*-methyl catalpol, acylated derivatives of catalpol, and linarin were isolated and identified. The anti-obesity activity of the extract and primary fractions was evaluated in a *Caenorhabditis elegans* model of obesity. Significant lipid-reducing activity was demonstrated in four fractions, indicating promising anti-obesogenic properties. Following chemical profiling and quantitative analysis, the main components of the most active fractions were identified, namely the *cis*- and *trans*-harpagoside isomers. Subsequent experiments demonstrated that treatment with harpagoside reduced lipid accumulation and improved mitochondrial function in glucose-supplemented worms, with the data suggesting potential involvement of the SKN-1 signaling pathway.

## 1. Introduction

Obesity is a complex metabolic disorder with increasing prevalence worldwide. As a significant burden on healthcare systems and a serious contributor to reduced quality and longevity of life, obesity has become an urgent topic in contemporary drug discovery field [1,2,3]. Plant extracts and their secondary metabolites hold particular promise in this context, largely due to their multimodal mechanism of action that modulates the complex pathophysiological mechanisms underlying excessive body weight [2,4].

Within modern natural product-based approaches targeting obesity, one well-established model system is *Caenorhabditis elegans*. While initially introduced over half a century ago as a model for neuroscience, this tiny nematode has since been widely applied in research on aging, metabolism, metabolomics, and natural products discovery. Advantages of *C. elegans* include its fully sequenced genome, the presence of evolutionarily conserved genes associated with human diseases, and a well-mapped neuronal network [3,5].

*Scrophularia aestivalis* Griseb., a Balkan endemic species, belongs to the genus *Scrophularia* (Scrophulariaceae), one of the most diverse genera within the family, comprising approximately 250 species [6]. This species is native to parts of Southeastern Europe, including Bulgaria, Albania, Greece and Serbia [7]. Representatives of genus *Scrophularia* have been used since ancient times as remedies for scrofula, scabies, tumors, eczema, psoriasis, and various inflammatory conditions. Several countries, including China, Korea, and Japan, employ these species as anti-inflammatory and anti-cancer agents. Roots of *S. ningpoensis* Hemsl., *S. buergeriana* Miquel, Ann., and *S. nodosa* L. are used to treat fever, swelling, constipation, pharyngitis, neuritis, and laryngitis. In Europe, *S. aquatica* L. has been used as a laxative, cardiac and circulatory stimulant, and diuretic, while the roots and aerial parts of *S. lucida* L. were used similarly in ancient Iranian medicine [8]. These species are included in the respective national pharmacopoeias, including those of China, the UK, Japan, and France.

The high therapeutic potential of *Scrophularia* species has driven extensive research on their chemical composition and pharmacological properties, leading to the isolation and characterization of over 200 individual compounds. These include iridoid and phenylethanoid glycosides, flavonoids, phenolic acids, triterpene saponins, and alkaloids [8], which exhibit diverse biological activities such as antioxidant, antimicrobial, antiviral, anti-inflammatory, enzyme-inhibiting, hepatoprotective, and immunoregulatory effects.

Despite this broad spectrum of biological activity, the potential of *Scrophularia* species for obesity management remains largely unexplored. To date, studies have reported lipase and α-amylase inhibitory activity of aqueous and methanol extracts of *S. ningpoensis* [9], and on α-amylase and α-glucosidase inhibition by fractions from *S. tenuipes*, a species endemic to Tunisia and Algeria [10]. Phytochemical and biological (in vitro) studies have attributed the enzyme inhibitory activity primarily to phenylethanoid [11]; however, other reports have linked such activity to the presence of iridoid glycosides, a class of compounds widely distributed in the Scrophulariaceae family [12]. Despite extensive research on the phytochemical composition of several *Scrophularia* species [8,13], *S. aestivalis*, native to Bulgaria, has not yet been phytochemically profiled, and its biological effects remain unexplored.

In this context, the present study aimed to investigate the phytochemical composition and anti-obesogenic activity of the previously unexplored *S. aestivalis*. To obtain a comprehensive understanding of its chemical composition, individual compounds were isolated and structurally characterized, and UPLC-HRMS/MS-based profiling was performed. The anti-obesogenic potential of the crude methanolic extract, fractions, and the main constituents was evaluated in a *C. elegans* model. Such an integrated and stepwise approach, from crude extract to individual isolated compounds, allows robust chemical characterization and the identification of bioactive compounds, and enables a better understanding of the therapeutic potential of the plant.

## 2. Results

### 2.1. Isolation and Identification by NMR. UPLC-HRMS/MS Profiling

**Figure 1:**
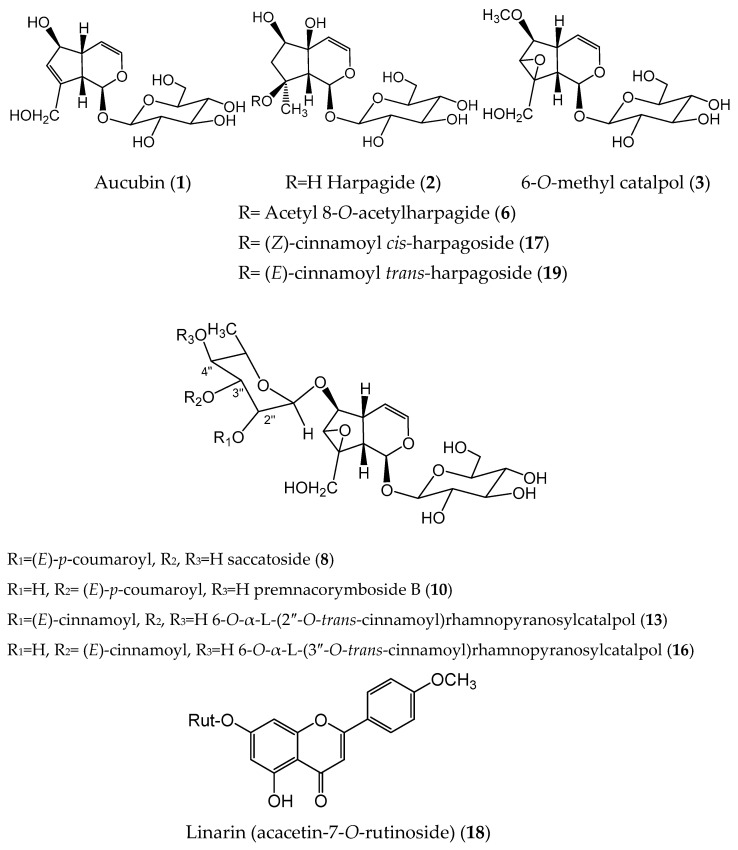
Structures of the compounds isolated from methanolic extract of *Scrophularia aestivalis*.

The methanol extract of *S. aestivalis* (SCA) was fractionated to obtain 11 crude fractions (SCA-1A-1K). As a result of subsequent separation and purification of fractions SCA-1C, SCA-1D, SCA-1E, and SCA-1J, ten known C-9 iridoid glycosides and one flavone-*O*-glycoside (Figure 1) were isolated and identified by comparison of their NMR spectral data with those reported in the literature: aucubin (**1**) [14], harpagide (**2**) [15], 6-*O*-methyl catalpol (**3**) [16], 8-*O*-acetyl harpagide (**6**) [17], saccatoside (**8**) [18], premnacorymboside B (**10**) [19] 6-*O*-α-L-(2″-*O*-*trans*-cinnamoyl)rhamnopyranosylcatalpol (**13**) [20], 6-*O*-α-L-(3″-*O*-*trans*-cinnamoyl)rhamnopyranosylcatalpol (**16**) [20], *cis*-harpagoside (**17**) [21], *trans*-harpagoside (**19**) [15], and linarin (acacetin-7-*O*-rutinoside **18**) [22].

Compounds **1**, **2**, **3**, **6**, **17**, and **19** are typical for genus *Scrophularia*, while the iridoids **8**, **10**, **13**, and **16** were previously found in *Verbascum* ssp. [12]. Linarin (**18**) is characteristic of *Budlleja* and *Linaria* species [23].

**Figure 2:**
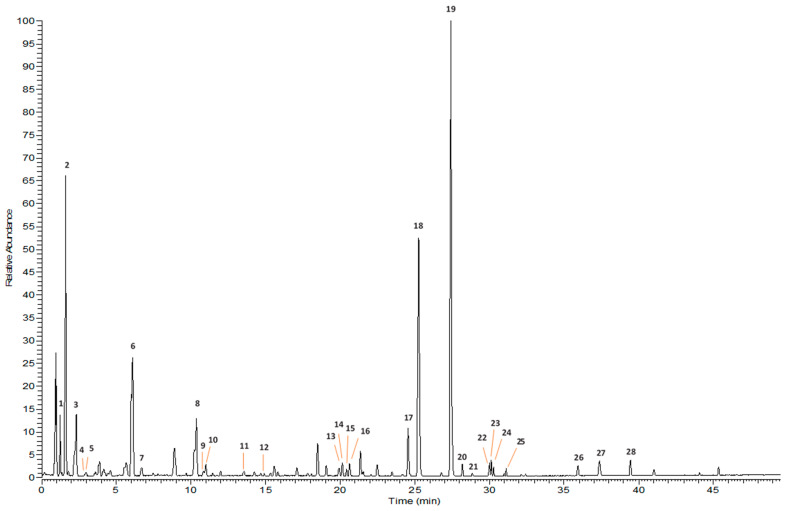
UPLC-HRMS/MS base peak chromatographic profile of SCA extract in negative ionization mode.

To obtain a complete phytochemical profile of the extract, ultra-high liquid chromatography with high-resolution mass spectrometry (UPLC-HRMS/MS)-based analysis was conducted in negative ion mode. Table 1 provides a summary of the retention times, molecular formulas, exact masses, delta (Δ) mass error in ppm, names of identified compounds, and proposed names of tentatively identified compounds. The number of assigned compounds corresponds to the elution order and to those indicated in Figure 1 and Figure 2.

The isolated compounds were used as standards, and several minor components were tentatively identified by comparison of their spectral data with those reported in the literature. Based on their molecular ion peaks [M+HCOO]^−^ or [M-H]^−^ and the characteristic ions in the MS/MS spectra, *cis*- and *trans*-caffeic acid, *p*-coumaric acid, two isomers of saccatoside (compounds **4**, **5**, **7**, **9** and **11**) [13], verbascoside, and *p*-coumaroyl harpagide (**12** and **15**) [24] were also detected. Extraction of the diagnostic ions at *m*/*z* 309.0985, 163.03993, and 145.0285 suggested that compound **14** is a *p*-coumaroyl-glucoside derivative, while the diagnostic ion at *m*/*z* 147.0443 indicated that compounds **23** and **24** are cinnamoyl derivatives [13,24,25].

**Table 1:** UPLC-HRMS/MS mass spectrometric data of tentatively identified compounds in *S. aestivalis* methanolic extract.

No. ^a^	Rt ^b^, min	MF ^c^	Exp. *m*/*z* [M-H], [M+HCOO]^−^	Calculated Mass	Δ Mass, ppm	MS/MS Product Ions [*m*/*z*]	Identification	Reference
**1**	1.22	C_15_H_22_O_9_	391.1246	346.1246	1.76	183.0656, 165.0549, 139.0549, 119.0549, 89.0229	Aucubin	Std.
**2**	1.61	C_15_H_24_O_10_	409.1351	364.1369	0.76	201.0764, 183.0657, 165.0554, 179.0556, 119.0339	Harpagide	Std.
**3**	2.33	C_16_H_24_O_10_	421.1359	376.1369	1.70	183.0663, 213.0769, 195.0657, 163.0395, 113.0239	6-*O*-Methyl catalpol	Std.
**4**	4.11	C_9_H_8_O_4_	179.0343	180.0422	−4.09	134.9868, 90.9968	*cis*-Caffeic acid	[13]
**5**	4.17	C_9_H_8_O_4_	179.0342	180.0422	−4.23	134.9869, 90.9968	*trans*-Caffeic acid	[13]
**6**	6.08	C_17_H_26_O_11_	451.1462	406.1475	1.03	301.6125, 183.0656, 165.0557, 119.0337	8-*O*-*acetylharpagide*	Std.
**7**	6.71	C_9_H_8_O_3_	163.0392	164.0473	−4.76	119.0491	*p*-Coumaric acid	[13,25]
**8**	10.37	C_30_H_38_O_16_	653.2102	654.2160	−3.56	377.1252, 325.8607, 315.1097, 309.0978, 291.0882, 187.0396, 181.0497, 163.0394, 145.0286, 119.0491	Saccatoside	Std.
**9**	10.82	C_30_H_38_O_16_	653.2102	654.2160	−3.54	377.1250, 325.8607, 315.1096, 309.0977, 291.0883, 187.0394, 181.0498, 163.0394, 145.0286, 119.0492	*p*-Coumaroyl rhamnopyranosylcatalopol isomer	
**10**	11.00	C_30_H_38_O_16_	653.2101	654.2160	−4.36	377.1250, 325.8607, 315.1096, 309.0977, 291.0883, 187.0394, 181.0498, 163.0394, 145.0286, 119.0492	Premnacorymboside B	Std.
**11**	13.54	C_30_H_38_O_16_	653.2100	654.2160	−3.89	377.1250, 325.8607, 315.1096, 309.0977, 291.0883, 187.0394, 181.0498, 163.0394, 145.0286, 119.0492	*p-Coumaroyl rhamnopyranosylcatalopol isomer*	
**12**	14.69	C_29_H_36_O_15_	623.1993	624.2054	1.92	461.1671, 161.0236, 113.0233	Verbascoside	[24,25]
**13**	19.92	C_30_H_38_O_15_	683.2205	638.2211	1.46	361.1303, 215.0710, 163.0392, 147.0442, 113.0233	6-*O*-*α*-L-(2”-*O*-*trans*-Cinnamoyl) rhamnopyranosylcatalpol	Std.
**14**	20.15	C_35_H_32_O_12_	689.1869	644.1894	−0.69	309.0985, 187.0394, 163.0392, 145.0285, 119.0491	*p*-Coumaroyl glycoside	[13]
**15**	20.43	C_24_H_30_O_12_	509.1671	510.1737	1.25	201.0764, 183.0654, 163.0392, 145.0285, 119.0491	*p*-Coumaroyl harpagide	[24]
**16**	20.65	C_30_H_38_O_15_	683.2205	638.2211	0.87	361.1297, 215.0709, 163.0391, 147.0441, 113.0239	6-*O*-*α*-L-(3”-*O*-*trans*-Cinnamoyl) rhamnopyranosylcatalpol	Std.
**17**	24.55	C_24_H_30_O_11_	539.1775	494.1788	−0.88	183.0658, 165.0549, 147.0442, 103.0540	*cis*-Harpagoside (8-*O*-(*Z*)-Cinnamoylharpagide)	Std.
**18**	25.26	C_28_H_32_O_14_	637.1788	592.1792	1.26	283.0616, 162.446	Linarin (Acacetin-7-*O*-rutinoside)	Std.
**19**	27.40	C_24_H_30_O_11_	539.1778	494.1788	−1.45	207.0663, 183.0656, 165.0580, 147.0443, 139.0391, 113.0233	*trans*-Harpagoside (8-*O*-(*E*)-Cinnamoylharpagide)	Std.
**20**	28.20	C_30_H_34_O_15_	679.1894	634.1897	−1.45	283.0616, 193.5697	Linarin derivative	
**21**	28.86	C_25_H_32_O_12_	523.1829	524.1893	1.46	274.2028, 174.9554, 154.0627, 147.0442, 130.1828, 119.5446	unknown	
**22**	29.98	C_30_H_34_O_15_	679.1895	634.1897	−1.37	283.0616, 193.5697	Linarin derivative	
**23**	30.10	C_25_H_28_O_11_	503.1780	504.1788	2.42	323.0932, 209.0969, 175.0397, 147.0443, 131.0492	Cinnamoyl derivative	[13]
**24**	30.27	C_25_H_30_O_11_	505.1725	506.1788	1.94	299.1295, 195.0658, 147.0443, 133.0649, 113.0233	Cinnamoyl derivative	[13]
**25**	31.12	C_18_H_32_O_5_	327.2183	328.2249	1.91	174.9924, 125.8304, 98.9845	unknown	
**26**	35.91	-	989.5344	-	-	811.4868, 649.4336, 471.3470, 161.0447, 191.0559, 143.0339, 127.9389	unknown	
**27**	37.38	C_16_H_12_O_5_	283.0613	284.0684	−1.69	179.2318, 112.2200, 71.4901	Acacetin	
**28**	39.45	-	957.5082	-	-	617.4067, 161.0445, 145.0497, 101.0231	unknown	

^a^ Numbers of the assigned compounds correspond to the elution order, ^b^ Rt—retention time, ^c^ MF—molecular formula; Std—isolated and identified compound used as standard for UPLC-MS/MS analysis.

### 2.2. The SCA Fractions Modulate Lipid Accumulation in a Glucose-Induced Obesity Model

**Figure 3:**
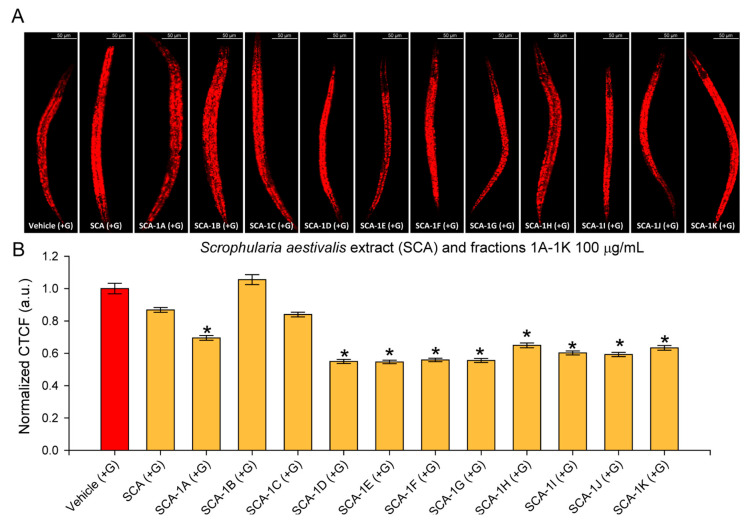
Effect of *S. aestivalis* extract (SCA) and its primary fractions 1A-1K on fat accumulation in *C. elegans* glucose-induced (+G) obesity model. (**A**) Representative confocal images (20× magnification, scale bar: 50 μm) of glucose-fed *C. elegans* supplemented with 100 μg/mL of SCA or its fractions (1A-1K). (**B**) Quantification of lipid accumulation. Fluorescence intensity for each worm was calculated as Correlated Total Cell Fluorescence (CTCF) using the formula: CTCF = Integrated Density − (Area × Mean background fluorescence). Values were normalized to the mean CTCF of the vehicle-treated (+G) group. Data are presented as mean ± SEM, (n = 90 worms from three independent biological replicates). * *p* < 0.05 vs. vehicle (+G) using ANOVA on ranks with Dunn’s post hoc test.

Despite the growing interest in the pharmacological potential of *Scrophularia* species, no information has been reported to date regarding the effect of *S. aestivalis* on lipid metabolism. To investigate whether the SCA extract or its fractions could alter fat storage, we employed a glucose-induced lipid accumulation model in *C. elegans*. For lipid quantification, Nile red staining was utilized as a widely used method for the visualization of neutral lipids in worms. While this approach has certain limitations, such as interference from gut autofluorescence and potential staining artefacts, it serves as a valuable preliminary tool for assessing the anti-obesogenic potential of natural products in *C. elegans*. The results revealed that, while the crude SCA extract failed to reduce fat accumulation, most of its fractions, including 1A and 1D-1K, significantly decreased the triglyceride content, with the strongest effect observed in fractions 1D-1G (Figure 3A,B). These findings raised the question of whether the biological activity could be attributed to specific components within these four primary fractions.

### 2.3. Qualitative Analysis by UPLC-HRMS/MS of SCA Active Fractions

To address the possibility of compound-dependent biological activity, the most active fractions (SCA-1D-1G) were analyzed by the UPLC-HRMS/MS method applied to the total extract. Based on MS peak areas of individual compounds in ion chromatograms, *cis*- and *trans*-harpagoside (**17** and **19**) were found to be the most abundant compounds in all four fractions. In addition, two isomers of compound **13** and martynoside were detected [13] (Table S2, Figures S1 and S2 in Supplementary Material).

### 2.4. Quantitative Analysis by HPLC-UV of SCA Extract and Active Fractions

Since *cis*- and *trans*-harpagoside are the major compounds and likely key contributors to the anti-obesogenic activity of the fractions, we further focused on their quantitative determination. HPLC-UV was utilized and their presence in the studied extract and its fractions was confirmed by comparing the retention times (RT) and UV spectra with those of the isolated compounds used as reference standards. The target compounds showed HPLC peak purities of 90% for *cis*-harpagoside and 95% for *trans*-harpagoside. Additionally, the UV spectra of the harpagoside isomers revealed maxima at 267.02 and 334.29 nm for *cis*-harpagoside and 280.52 nm for *trans*-harpagoside, respectively, and all chromatograms were monitored at 275 nm.

Quantification was performed using six-point calibration curves (Figure S3) established for both *cis*-harpagoside (RT: 35.311 min y = 9155.9x − 19874; r^2^ = 0.996; range: 5.00–100.00 µg/mL) and *trans*-harpagoside (RT: 37.944 min; y = 11,000x − 57480; r^2^ = 0.999; range: 15.00–200.00 µg/mL). The limits of detection (LOD) and quantification (LOQ) for both compounds were calculated based on the standard deviation of the y-intercept of the regression line. The LOD and LOQ were estimated at 5.67 µg/mL and 17.20 µg/mL for *cis*-harpagoside, and 4.51 µg/mL and 13.67 µg/mL for *trans*-harpagoside, respectively. Furthermore, the method demonstrated peak resolution greater than 1.5 and peak symmetry close to 1.0, indicating high chromatographic efficiency and suitability for quantitative analysis (Supplementary Figure S4).

**Figure 4:**
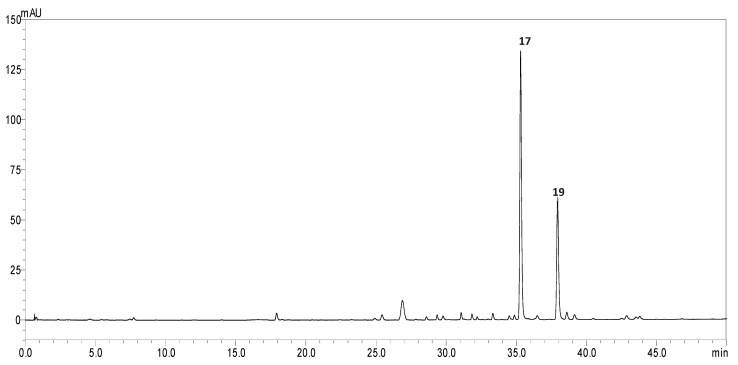
HPLC-UV chromatographic profile of total methanolic SCA extract at 275 nm, where **17** is *cis*-harpagoside and **19** is *trans*-harpagoside.

**Figure 5:**
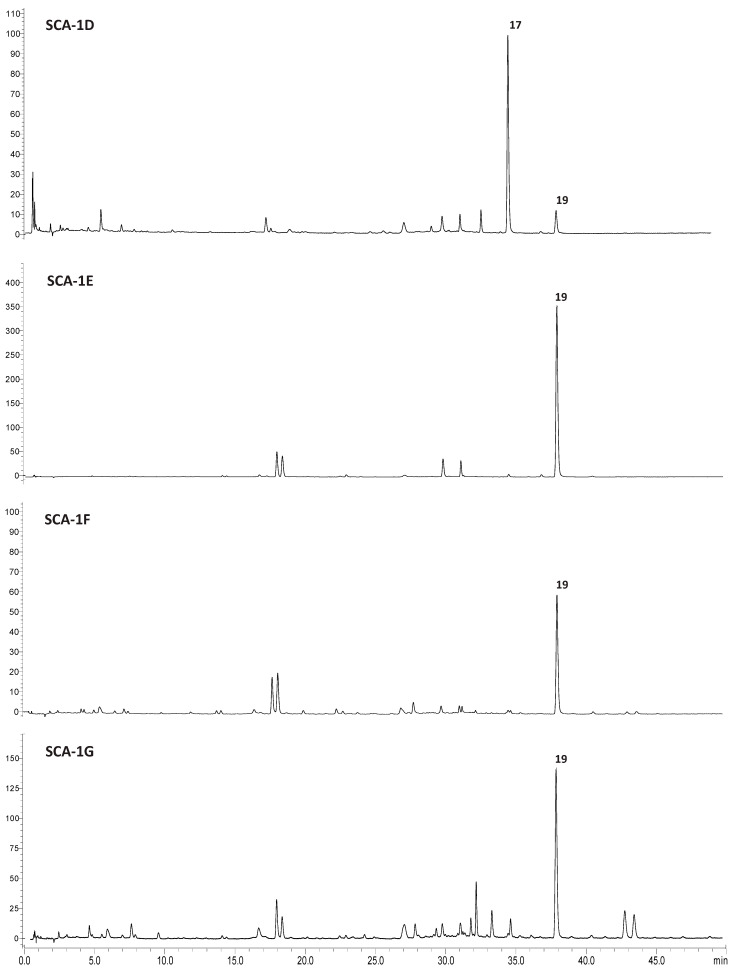
HPLC-UV chromatographic profiles recorded at 275 nm for fractions SCA-1D–1G, where **17** is *cis*-harpagoside and **19** is *trans*-harpagoside.

In conclusion, the method demonstrated selectivity for both studied compounds, exhibiting linearity (R^2^ > 0.99) across both studied concentration ranges, minimal deviation in intra- and inter-day precision (RSD < 2%), and acceptable LOD and LOQ values. Representative UV chromatographic profiles at 275 nm of the SCA extract and fractions SCA-1D to SCA1G are shown in Figure 4 and Figure 5, respectively.

Accordingly, the proposed HPLC was successfully applied to quantify the major iridoid glucosides both in the total extract and its active fractions. The quantitative analysis data are summarized in Table 2.

*Trans*-harpagoside was identified as the most abundant iridoid in fraction E with a concentration of 501.06 ± 0.33 μg/mg d. extr., followed by fraction G (121.45 ± 0.16 μg/mg d. extr.). A lower amount of *trans*-harpagoside was detected in the total SCA extract, measured as 45.17 ± 0.44 μg/mg d. extr. In contrast, *cis*-harpagoside was found to be the predominant iridoid in the total extract, with a concentration of 85.35 ± 0.42 μg/mg d. extr. The only fraction containing *cis*-harpagoside was fraction D (58.25 ± 0.53 μg/mg d. extr.), which showed a 27.1% lower amount of the compound compared to the total extract.

Based on these quantitative results, the concentrations of both isomers in the crude extract and in the four primary fractions (SCA-1D–1G) exhibiting the highest anti-obesogenic potential were recalculated to reflect their actual levels under. The concentrations of *cis*- and *trans*-harpagoside (expressed in μg/mL and μM) are presented in Table 3. Among all analyzed fractions, fraction E exhibited the highest level of *trans*-harpagoside, corresponding to approximately 100 μM at the tested concentration (100 μg/mL). Based on this finding, the same molar concentration was selected for the subsequent biological assays with both *cis*- and *trans*-harpagoside.

### 2.5. Anti-Obesogenic Effect of Isolated cis- and trans-Harpagoside in C. elegans

**Figure 6:**
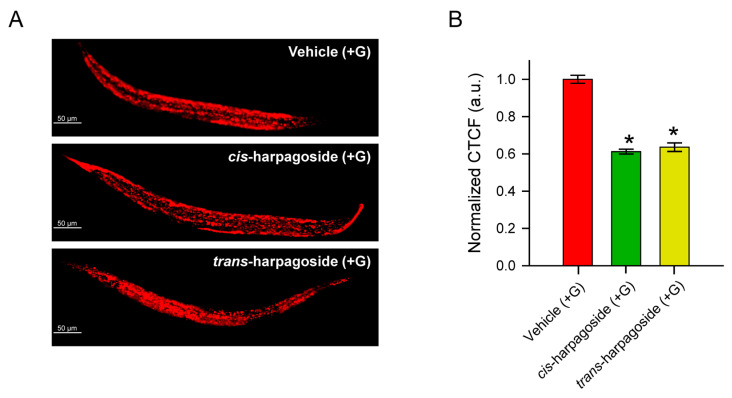
Effect of *cis*- and *trans*-harpagoside isomers on fat accumulation in *C. elegans*. (**A**) Representative confocal images (20× magnification, scale bar: 50 μm) of Nile red-stained *C. elegans* treated with 100 μM *cis*- or *trans*-harpagoside. (**B**) Quantification of lipid accumulation. Fluorescence intensity of each worm was calculated as Correlated Total Cell Fluorescence (CTCF) using the formula: CTCF = Integrated Density − (Area × Mean background fluorescence). Values were normalized to the mean CTCF of the vehicle (+G) group. Data are presented as mean ± SEM, (n = 90, from three independent biological replicates), * *p* < 0.05 vs. vehicle (+G) group using ANOVA on ranks with Dunn’s post hoc test.

Harpagoside is a naturally occurring iridoid glycoside with well-documented biological activities, including anti-inflammatory, anti-rheumatic, and cardioprotective effects. Some evidence also suggests that it may prevent obesity-induced atherosclerosis [26,27,28,29,30]. In this context, and given the fat-lowering activity of the four most active fractions, which are rich in harpagoside, we investigated whether the isolated *cis*- and *trans*-harpagoside isomers could modulate glucose-induced fat accumulation in *C. elegans*. The results revealed that both isomers significantly reduced lipid content (Figure 6A,B), strongly supporting the hypothesis that harpagoside is the principal bioactive constituent responsible for the lipid-lowering effect observed in these fractions.

### 2.6. Harpagoside Isomers Alleviate Glucose-Induced Mitochondrial Dysfunction in C. elegans

**Figure 7:**
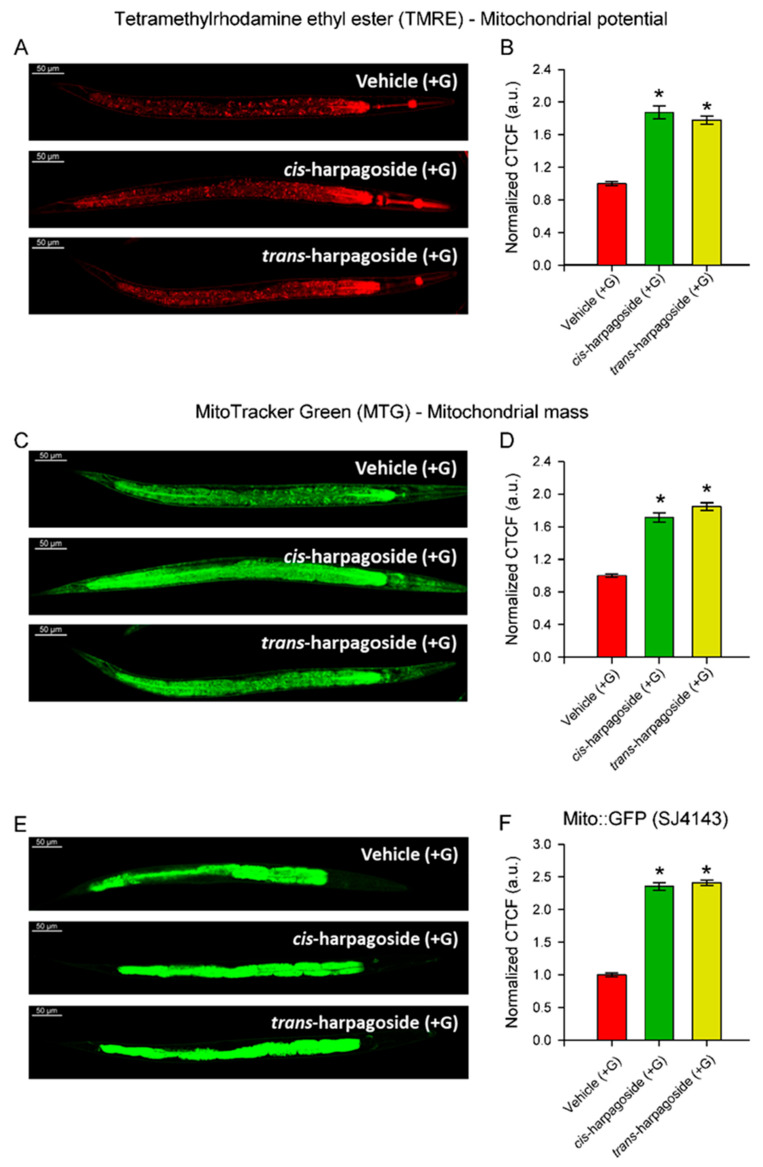
The two isomers of harpagoside improve mitochondrial health in a model of glucose (+G)-induced mitochondrial dysfunction. (**A**) Representative confocal images (20× magnification, scale bar: 50 μm) showing TMRE fluorescence (red channel) in glucose-fed N2 worms supplemented with 100 µM *cis*- or *trans*-harpagoside. (**B**) Quantification of mitochondrial membrane potential. Red channel fluorescence intensity was calculated for each worm as Correlated Total Cell Fluorescence (CTCF): CTCF = Integrated Density − (Area × Mean background fluorescence). Values were then normalized to the mean CTCF of the vehicle (+G)-treated group. Data are presented as mean ± SEM (n = 60 worms from three independent biological replicates). * *p* < 0.05 vs. vehicle (+G), ANOVA on ranks with Dunn’s post hoc test. (**C**) Representative confocal images (20× magnification, scale bar: 50 μm) showing green channel fluorescence (MTG) of glucose-fed N2 worms supplemented with *cis*- or *trans*-harpagoside. (**D**) Quantification of mitochondrial mass. Green channel fluorescence intensity calculated as CTCF and normalized to vehicle (+G) as in (B). n = 60, 3 replicates. * *p* < 0.05 vs. vehicle (+G), ANOVA on ranks with Dunn’s post hoc test. (**E**) Representative confocal images (20× magnification, scale bar: 50 μm) of SJ4143 zcIs17 [ges-1::GFP(mit)] worms treated with *cis*- or *trans*-harpagoside. (**F**) Quantification of GFP signal (green channel), calculated as CTCF and normalized to vehicle (+G). Data are presented as mean ± SEM (n = 60 worms from three independent biological replicates). * *p* < 0.05 vs. vehicle (+G), ANOVA on ranks with Dunn’s post hoc test.

Since both isomers significantly reduced lipid deposition in glucose-supplemented worms, we next examined whether this effect could be associated with improved mitochondrial function. Mitochondrial membrane potential and mass were evaluated using co-staining with Tetramethylrhodamine ethyl ester (TMRE) and MitoTracker Green (MTG), respectively. The assessment of mitochondrial membrane potential (Figure 7A,B) and mass (Figure 7C,D) revealed that both parameters were significantly increased following harpagoside treatment compared to the vehicle (+G) group. In addition, intestinal mitochondrial activity was analyzed in the transgenic strain SJ4143 (Figure 7E,F), which demonstrated a pronounced increase in mitochondrial mass, consistent with the results obtained from MTG staining (Figure 7A,B). Collectively, these findings indicate that both *cis*- and *trans*-harpagoside exhibit a similar capacity to improve mitochondrial health under conditions of glucose-induced mitochondrial dysfunction.

### 2.7. The Transcription Factor SKN-1 and Its Downstream Target gst-4 Are Upregulated upon cis- and trans-Harpagoside Treatment

The transcription factor skinhead-1 (SKN-1), the *C. elegans* ortholog of human nuclear factor erythroid 2-related factor 2 (NRF2), has been shown to play important role in regulating mitochondrial homeostasis, particularly under conditions of mitochondrial dysfunction. It is also crucial for metabolic adaptation, ensuring proper cellular function [3,31,32,33].

Based on our current experimental evidence that both isomers reduce lipid accumulation under glucose supplementation (Figure 6), as well as their effects on mitochondrial function in a model of glucose-induced dysfunction (Figure 7), we investigated whether SKN-1 could mediate these effects. To address this, we utilized the transgenic reporter strain LD1, which expresses SKN-1::GFP, allowing visualization and quantification of SKN-1 activity, along with its downstream target glutathione S-transferase 4 (*gst-4*) [3,5].

**Figure 8:**
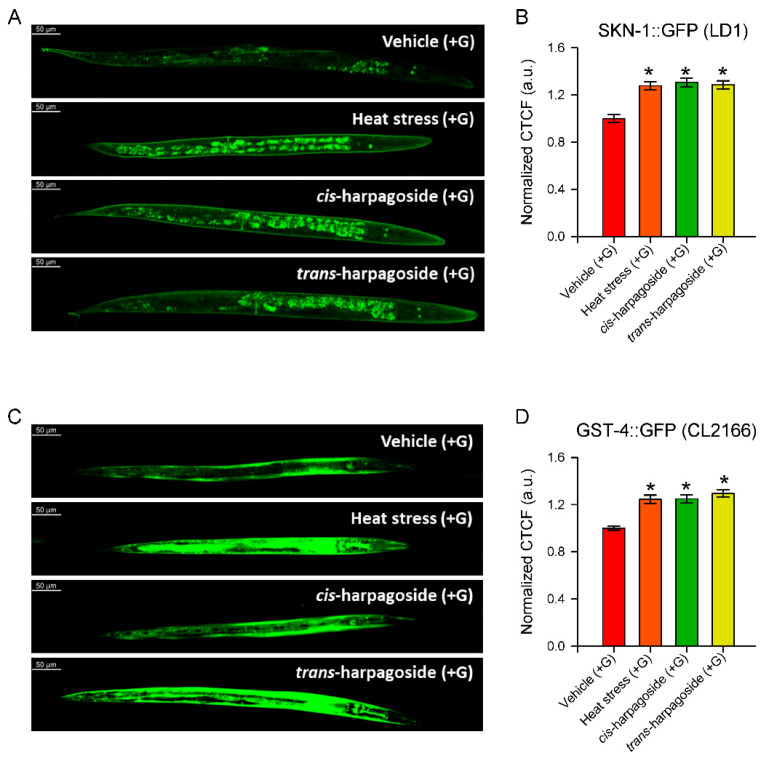
Upregulation of SKN-1 and *gst-4* in response to *cis*- or *trans*-harpagoside treatment. (**A**,**C**) Representative confocal fluorescence images (20× magnification, scale bar: 50 μm) of (**A**) LD1 and (**C**) CL2166 worms treated with vehicle (+G), exposed to heat stress (+G; 37 °C for 5 min), or supplemented with *cis*- or *trans*-harpagoside. (**B**,**D**) For each worm, fluorescence was calculated as Correlated Total Cell Fluorescence (CTCF): CTCF = Integrated Density − (Area × Mean background fluorescence). Values were normalized to the mean value of vehicle (+G) group and expressed in arbitrary units (a.u.). Data are presented as mean ± SEM (n = 60 worms from three independent biological replicates). * *p* < 0.05 vs. vehicle (+G), ANOVA on ranks with Dunn’s post hoc test.

Fluorescence analysis revealed that treatment with both *cis*- and *trans*-harpagoside significantly increased SKN-1 nuclear localization in LD1 nematodes (Figure 8A,B) and enhanced *gst-4* expression in the CL2166 strain (Figure 8C,D). These findings confirm our hypothesis that SKN-1 signaling is correlated with the lipid-lowering and mitochondrial-protective effects of both harpagoside isomers.

## 3. Discussion

Obesity is a global health burden, and its prevention and treatment are of great importance not only for individual well-being but also for the stability of healthcare systems worldwide [1,2]. Moreover, obesity is the major risk factor for the development of chronic diseases such as type 2 diabetes, cardiovascular disorders, and osteoarthritis, as well as for accelerated aging [1]. In the context of therapeutic approaches, numerous studies have highlighted the promising anti-obesity activity of both plant extracts and their secondary metabolites [3,34,35]. This underscores the relevance of exploring the broad spectrum of plant-derived secondary metabolites as a potential strategy for obesity management. In this regard, a key aspect of natural products research is the precise and robust characterization of extracts and compounds of interest.

A proper understanding of the anti-obesity potential of plant extracts and their fractions begins with analysis of their bioactive compounds using appropriate analytical methodologies. In the present study, a phytochemical composition of a previously unexplored *S. aestivalis* was investigated through chemical profiling of the total methanolic extract and its fractions, along with isolation of individual compounds. Ten C-9 iridoid glycosides and one flavone-*O*-glycoside were isolated and structurally characterized, while additional iridoids, phenolic acids and a phenylethanoid glycoside were tentatively identified. Among the compounds, two iridoid glycosides—*cis*- and *trans*-harpagoside—were detected as the major constituents of *S. aestivalis*, and a HPLC method was developed for their quantification. In parallel with extract characterization and the identification of its main secondary metabolites, the anti-obesogenic activity was evaluated in vivo using *C. elegans* model of glucose-induced lipid accumulation. The effects of SCA extract, its fractions, and both *cis*- and *trans*-harpagoside assessed. In addition, harpagoside, identified as the major compound in the four most potent fractions, was further tested for its protective potential on mitochondrial homeostasis and its modulatory effect on SKN-1, a transcription factor essential for cellular integrity, and its downstream target *gst-4*.

The family Scrophulariaceae, commonly known as figworts, is well recognized for its diverse range of biological activities, primarily attributed to its rich content of iridoids. In plants, iridoids play an important protective role, while in humans and animals they have been associated with anti-inflammatory, antidiabetic, hepatoprotective, neuroprotective, wound-healing, and antiviral effects [36,37,38]. With regard to metabolic regulation, several *Scrophularia* species are important components of anti-obesity and antihypertensive herbal formulations. An example is the dried roots of *S. ningpoensis*, which are traditionally used in Chinese and Vietnamese medicine [39,40,41]. However, *Scrophularia* species from the Balkan region, including *S. aestivalis*, have not been sufficiently evaluated for their effect on metabolic disorders [42].

In the present study, treatment with the crude SCA extract did not reduce triglyceride content at 100 μg/mL in glucose-fed nematodes. The absence of lipid-lowering activity may be related to matrix effects [43,44]. In contrast, both methanolic and aqueous extracts of *S. ningpoensis* have been reported to inhibit pancreatic lipase and α-amylase, with stronger activity observed for the methanolic extract [9]. Such discrepancies could arise from species-specific differences or from the distinct experimental models employed. Interestingly, four primary SCA fractions exhibited marked lipid-reducing activity at the same supplementation dose (100 μg/mL). Metabolite profiling further indicated that harpagoside was the predominant constituent detected in the fractions.

Harpagoside is well known for its potential in the treatment of chronic non-communicable diseases such as arthritis, in which dysregulated inflammatory responses play a central role [27,45]. In addition, harpagoside has been shown to exert anti-osteoporotic activity by modulating bone Morphogenetic Protein 2 and Wnt signaling in osteoblasts, while inhibiting osteoclast differentiation [46]. A study investigating its anti-obesity potential in 3T3-L1 adipocytes showed that harpagoside inhibited tumor necrosis factor-α (TNF-α)-induced adipokine expression by activating of peroxisome proliferator-activated receptor gamma (PPAR-γ) [47]. Other structurally related iridoids from *Scrophularia* species have also shown protective effects in various pathological contexts. For instance, harpagide, a major constituent of *S. ningpoensis*, was reported to protect rat cortical neurons subjected to oxygen-glucose deprivation and reoxygenation, a model of cerebral ischemia–reperfusion injury, by attenuating endoplasmic reticulum stress [48]. As the 8-*O*-cinnamoyl ester of harpagide, harpagoside may share, and potentially enhance, some of these protective properties.

Our results suggest that both *cis*- and *trans*-harpagoside exhibit comparable anti-obesogenic activity at the tested concentration (100 μM). Quantitative analysis of harpagoside in SCA and its fractions revealed that the extent of lipid-reducing activity did not directly correspond with the measured harpagoside content. In fraction 1E, the concentration of *trans*-harpagoside was similar to that used in the assays with both isolated isomers, whereas other active fractions contained lower levels or different isomer ratios. These observations suggest that overall biological activity may be influenced by the complex matrix composition, potentially through interactions among multiple constituents leading to synergistic effects [49]. Interestingly, an extract from another source of harpagoside, *Harpagophytum procumbens*, has been reported to suppress appetite via activation of growth hormone secretagogue receptor (GHS-R1a), although the harpagoside itself did not exhibit similar activity [50].

Glucose supplementation not only alters lipid metabolism, leading to excessive triglyceride accumulation in *C. elegans*, but also compromises mitochondrial health [51]. In obesity, mitochondrial homeostasis is typically deteriorated, with energy metabolism playing a central role in these processes [3,31]. To the best of our knowledge, this is the first report demonstrating that both isomers of harpagoside can improve mitochondrial function in a glucose-induced model of obesity in *C. elegans*. Previous studies have also suggested beneficial effects of harpagoside on mitochondria, specifically regarding the restoration of mitophagy flux and the reduction in mitochondrial oxidative stress through modulation of key proteins involved in mitophagy, such as PINK 1 and Parkin [30]. In another study, harpagoside counteracted rotenone-induced mitochondrial impairment in cellular models of Parkinson’s disease by restoring complex I activity and alleviating mitochondrial swelling [52].

In our experimental model, glucose supplementation is known to induce negative mitochondrial changes—including disrupted morphology, reduced content, and decreased membrane potential—which have been closely associated with muscle degradation, impaired ATP production, accelerated aging, and increased susceptibility to stress in *C. elegans* [53,54,55]. Here, we demonstrate that both harpagoside isomers enhance mitochondrial mass and membrane potential, thereby mitigating glucose-induced mitochondrial dysfunction.

The transcription factor SKN-1, the *C. elegans* ortholog of human NRF2, plays an essential role in regulating mitochondrial dynamics, particularly under conditions of mitochondrial dysfunction. It is also crucial for metabolic adaptation and the maintenance of cellular homeostasis [31,32,33]. Additionally, NRF2 is typically associated with oxidative stress response, while acting interdependently with other mediators of cellular adaptation. On the other hand, mechanistic studies investigating the activity of harpagoside have mainly focused on inflammation-related pathways, including cytokines such as interleukin-6 and TNF-α, and transcription factors such as c-FOS, c-Jun, nuclear factor kappa-light-chain-enhancer of activated B cells (NF-κB), CCAAT/enhancer-binding protein β (C/EBPβ) [28,56].

We demonstrate that both *cis*- and *trans*-harpagoside activated SKN-1 and its downstream target *gst-4* in transgenic reporter strains, suggesting that the metabolic effects of these isomers may at least in part involve this conserved stress-response pathway. Supporting this, treatment with *H. procumbens* extract, have been shown to increase NRF2 levels in neurons, thus reducing oxidative stress [56]. However, further mechanistic studies are needed to confirm and extend the hypothesis regarding the involvement of this signaling pathway.

## 4. Materials and Methods

### 4.1. General Experimental Procedures

For the qualitative analysis Q Exactive Plus^®^ hybrid quadrupole-Orbitrap^®^ mass spectrometer (HRMS/MS) equipped with heated electrospray ionization source (HESI) coupled with a Vanquish UPLC system (Thermo Fisher Scientific, Bremen, Germany) was used. The quantitative analysis was conducted using a Shimadzu (Nexera-LC40 XS, Kyoto, Japan) chromatograph equipped with a vacuum degasser (DGU-405), a binary pump (LC-40D XS), an autosampler (SIL-40C XS), a column oven (CTO-40C) and a PDA detector (M40). Final purification of the isolated compounds was carried out using Pure C-850 FlashPrep chromatography system with integrated UV and ELSDs and a glass column FP Select 230 × 15 mm (Buchi, Uster, Switzerland) (C18 Silica Gel, 20 g, 230 × 15 mm). The NMR spectra were recorded on Bruker AVANCE NEO 400 and Bruker AVANCE NEO 600 spectrometers (Biospin GmbH, Rheinstetten, Germany). The NMR spectra of compounds were performed in CH_3_OH-*d*_4_ purchased from Deutero-GmbH (Kastellaun, Germany). The spectra are processed with the Topspin 4.1.4 program.

### 4.2. Caenorhabditis Elegans Maintenance and Treatment

The N2 wild-type strain (Bristol), LD1 ldIs7 [skn-1b/c::GFP + rol-6(su1006)], CL2166 dvIs19 [(pAF15)gst-4p::GFP::NLS] III, SJ4143 zcIs17 [ges-1::GFP(mit)] of *C. elegans* and *Escherichia coli* OP50 were obtained from the Caenorhabditis Genetics Center (CGC, University of Minnesota, MN, USA), which is funded by the NIH Office of Research Infrastructure Programs (P40 OD010440). Nematodes were cultured at 20 °C under standard laboratory conditions on Nematode Growth Medium (NGM) agar plates seeded with *E. coli* OP50.

For the assays, a glucose was added to the NGM to a final concentration of 2% [3,34]. Synchronized worm populations were obtained using a standard hypochlorite bleaching method [5]. For the experimental treatments, *E. coli* OP50 was heat-inactivated by incubation at 65 °C for 30 min and subsequently concentrated tenfold by centrifugation. The test substances were added directly to the inactivated bacterial suspension, which was then seeded on the NGM plates.

Following synchronization, the worms were transferred onto the supplemented plates. The treatments included the crude extract (SCA), its fractions (1A to 1K), each applied at a final concentration of 100 μg/mL, and the isolated pure compounds *cis*- and *trans*-harpagoside, at a final concentration of 100 μM. The control group received 0.2% DMSO as a vehicle treatment.

### 4.3. Chemicals and Reagents

For fractionation of the extract and separation of prime fractions column chromatography (CC) was performed on Polyamide 6 (Sigma-Aldrich, St. Luis, MO, USA) and Sephadex LH-20 (25–100 µm, Pharmacia Fine Chemicals, Uppsala, Sweden). Detection of separation was achieved by thin layer chromatography (TLC) on 60 F254 plates (Merck, Darmstadt, Germany) under UV light at 254 and 366 nm and spraying with 20% (*v*/*v*) H_2_SO_4_ in EtOH solution. For isolation and purification of the individual compounds flash chromatography C18 Silica Gel, 20 g Merck, Darmstadt, Germany) was used. All solvents used for chromatographic purposes were of analytical grade. Acetonitrile and methanol (LC-MS Chromasolv-grade), used for LC-HRMS/MS analysis, were supplied by Honeywell-Riedel-de Haen (Seelze, Germany). Formic acid (97.5–98.5% purity, LC-MS Lichropur™, CAS: 64-18-6) was obtained from Sigma-Aldrich (Buchs, Switzerland). Acetonitrile and methanol (HPLC-grade), used for LC-UV analysis, were purchased from Sigma-Aldrich (Steinheim, Germany). Deionized water with a resistivity of ≤0.55 μS/cm was produced using a Smart2Pure 12UV/UF water purification system (Thermo Electron LED GmbH, Langenselbold, Germany).

Nematode Growth Medium (NGM; Cat. No. MBS652667) was obtained from MyBiosource Inc. (San Diego, CA, USA). Agar powder (Cat. No. 05039), LB Broth Lennox (Cat. No. L3022), and M9 Minimal Salts (Cat. No. M6030) were purchased from Sigma-Aldrich (St. Louis, MO, USA). Fluoroshield histology mounting medium (Cat. No. F6182) and Nile red (NR; Cat. No. 72485) were also obtained from Sigma-Aldrich (St. Louis, MO, USA). Tetramethylrhodamine, ethyl ester (TMRE, Cat. No. 11560796) was supplied from Invitrogen. MitoTracker Green FM (MTG, Cat. No. HY-135056) was purchased from MedChemExpress (Sollentuna, Sweden).

### 4.4. Plant Material

Aerial parts of *S. aestivalis* were collected during flowering period of the species in June 2023 from Rila Mt. North of Padala village, Rila municipality 1750 m alt. (GPS 28 June 2023, 42.16381° N, 23.18497° E). Voucher specimen (SOM 179574) has been deposited in the Herbarium of Institute of Biodiversity and Ecosystem Research, BAS.

### 4.5. Extraction Procedure and Isolation

The air-dried and grounded plant material (120 g) was extracted with 100% MeOH (2 L) through maceration at room temperature (2 × 48 h). Then, crude methanol extracts were combined, filtered and concentrated to dryness (11.5 g). Part of obtained extract (8.3 g) was dissolved in distilled H_2_O and subjected to column chromatography on Polyamide 6 eluted gradually with distilled H_2_O (500 mL), 25% MeOH (400 mL), 50% MeOH (450 mL), 75% MeOH (500 mL) and 100% MeOH (700 mL), to afford eleven main fractions (SCA-1A–SCA-1K). For further separation and isolation of secondary metabolites from the remaining fractions, Sephadex LH-20 column chromatography was performed using methanol (MeOH) as the eluent. Additionally, flash chromatography was applied using a mobile phase consisting of water (Solvent A) and methanol (Solvent B), with the following elution gradient: 0–60 min, 0 → 70% B; 60–65 min, 70 → 100% B; and 65–75 min, 100% B. During the analysis, the flow rate was maintained at 10 mL/min. Detection was performed using an Evaporative Light Scattering Detector (ELSD), and UV absorbance was monitored at multiple wavelengths: 205, 254, 280, and 350 nm. Thus, a part of SCA-1C (350 mg) was dissolved in MeOH and applied on Sephadex column and a subfraction (180 mg) was additionally separated by flash chromatography to afford aucubin (**1**, 14.2 mg), harpagide (**2**, 18.0 mg), 6-*O*-methyl catalpol (**3**, 12.1 mg) and 8-acetyl harpagide (**6**, 18.8 mg). Fraction SCA-1D (132 mg) was directly subjected on flash chromatography and as result 17.2 mg of pure *cis*-harpagoside (**17**, 17.0 mg) was isolated. 350 g of SCA-1E were also applied on Sephadex LH-20 column and the obtained two subfractions a_1_ (145 mg) and a_2_ (118 mg) were additionally separated by flash chromatography.

As result, saccatoside (**8**, 14.1 mg), premnacorymboside B (**10**, 14.0 mg) and *trans*-harpagoside (**19**, 31.0 mg), 6-*O*-α-L-(2″-*O*-(E)-cinnamoyl)rhamnopyranosylcatalpol (**13**, 4.7 mg), 6-O-α-L-(3″-*O*-(*E*)-cinnamoyl)rhamnopyranosylcatalpol (**16**, 5.5 mg) were isolated. Approximately 1 g of SCA-1J was dissolved in MeOH and subjected on Sephadex column and a subfraction (68 mg) was purified to obtaining of 13.7 mg linarin (**18**).

### 4.6. Qualitative Analysis by UPLC-HRMS/MS

LC-MS analysis was conducted using a high-resolution Q Exactive Plus^®^ hybrid quadrupole-Orbitrap^®^ mass spectrometer (HRMS/MS) equipped with HESI coupled with a Vanquish UHPLC system (Thermo Fisher Scientific, Bremen, Germany). Chromatographic separation of *S. aestivalis* compounds was achieved using an Accucore™ C18 analytical column (150 × 2.1 mm, 2.6 µm; Thermo Fisher Scientific™, Germany). The mobile phase comprised water with 0.1% (*v*/*v*) formic acid (Solvent A), and acetonitrile with 0.1% (*v*/*v*) formic acid (Solvent B). The flow rate was maintained at 0.3 mL/min, and the injection volume was 3 µL. Thus, the compounds’ elution occurred over a 50-min period with fallowing gradient program: 0–25 min, 7 → 22% % B; 25–30 min, 22 → 30% B; 30–40 min, 30 → 35% B; 40–50 min, 35 → 50% B; 50–55 min, 50 → 85% B; 55–60 min, 95 → 7% B, followed by 10-min re-equilibration to initial conditions. The operating conditions for the HESI source were as follows: 2.90 kV spray voltage; 320 °C capillary temperature; sheath gas flow rate, 30 arb. units; auxiliary gas flow, 6 arb. units; sweap gas flow, 0 arb. units, S-Lens RF level, 50 V. Full-scan mass spectra over the range 120–1200 were acquired in negative and positive ionization mode at resolution settings of 70,000, automatic gain control (AGC) target of 1e6, and a maximum injection time (IT) of 80 ms. Data acquisition and processing were carried out using Xcalibur software 4.2 SP1 and FreeStyle 1.5 (Thermo Fisher Scientific). The tentative identification of the phytochemicals was assigned in negative ion mode as [M-H]^−^ and [M+HCOO]^−^.

### 4.7. Quantitative Analysis by HPLC-UV

Quantitative analyses were conducted using a Shimadzu (Nexera-LC40 XS, Kyoto, Japan) chromatograph equipped with a vacuum degasser (DGU-405), a binary pump (LC-40D XS), an autosampler (SIL-40C XS), a column oven (CTO-40C) and a PDA detector (M40). Chromatographic separation of *S. aestivalis*’ constituents was carried out using an Accucore™ C18 analytical column (150 × 2.1 mm, 2.6 µm; Thermo Fisher Scientific™, Germany) using a 0.5 mL/min flow rate, a 5-μL injection volume, and a 40 °C oven temperature. The mobile phase encompassed water with 0.1% (*v*/*v*) formic acid (Solvent A), and 60% (*v*/*v*) acetonitrile in 0.1% (*v*/*v*) formic acid (Solvent B). Using the selected mobile phases, the following gradient was chosen as optimal for profiling all extract compounds: 0–25 min, 7 → 22% B; 25–30 min, 22 → 30% B; 30–40min, 30 → 35% B; 40–50 min, 35 → 50% B; 50–55 min, 50 → 85% B; 55–60 min, 95 → 7% B, followed by 10-min re-equilibration to initial conditions. Prior the analysis the mobile phases were filtered through an Olimpeak™ Nylon membrane filter (0.25 µm pore size, 47 mm diameter, Teknokroma Analitica SA, Barcelona, Spain). During the analysis, the autosampler temperature was maintained at 8 °C. The photodiode array (PDA) detector was configured to scan across a wavelength range of 200–700 nm (±0.01 nm), with peak areas quantified at 275 nm. Data acquisition and processing were carried out using LabSolutions software DB version 5.114 (Shimadzu, Kyoto, Japan).

In addition, method linearity for *cis*- and *trans*-harpagoside was established by correlating peak area with the concentration of standard solutions (μg/mL). System suitability was assessed by comparing chromatograms of mobile phase blanks with those of sample solutions with no co-eluting impurities. Method precision was evaluated through repeated injections of six standard solutions for intra-day analysis, and across three separate days for inter-day analysis.

### 4.8. LC Sample Preparation

For quantitative analysis, 3 mg of the dry *S. aestivalis* extract was accurately weighed and dissolved in 100% MeOH. From a starting concentration of 2 mg/mL, a volume of 500 μL was diluted to 1.5 mL with mobile phase A.

For qualitative analysis, appropriate quantities of the total SCA dry extract were purified using a solid-phase extraction (SPE) procedure with Supelco™ Superclean LC-18 tubes (3 μm particle diameter), purchased from Sigma-Aldrich (Taufkirchen, Germany). The final eluate was diluted to 10 mL with an 80% methanol-in-water solution, yielding a final concentration of 540 μg/mL.

The SCA fractions were prepared as follows: 1 mg of each dried fraction (1D, 1E, 1F, and 1G) was dissolved in 1 mL of 100% methanol. Subsequently, 500 μL of each stock solution was diluted with 1 mL of mobile phase A prior to analysis.

The stock standard solution was prepared by individually weighing appropriate amounts of *cis*- and *trans*-harpagoside, followed by dissolution in 100% methanol. Aliquots of this solution containing both standard compounds were subsequently diluted with mobile phase A to achieve the concentrations previously established for the construction of calibration curves for each compound.

All sample and standard solutions were filtered through Olimpeak™ PTFE membrane syringe filters (25 mm diameter; 0.20 μm pore size), obtained from Teknokroma Analitica SA (Barcelona, Spain), and stored at 10 °C in the LC autosampler prior to injection into the column.

### 4.9. Nile Red Triglyceride Staining

After exposure to the experimental treatments for 24 h, approximately 1000–1500 worms (L3–L4 larval stage) per experimental group were collected and washed three times with M9 buffer. Each sample was then fixed with 600 μL of 40% isopropanol for 3 min, followed by centrifugation and removal of the supernatant. Nile red staining solution was added to the pellet, and the samples were incubated for 2 h. After staining, the samples were centrifuged, the dye was removed, and the worms were incubated in PBST (phosphate-buffered saline with 0.1% Tween™ 20) for 30 min. The stained nematodes were mounted on glass microscope slides using a histology mounting medium [3,5]. Lipid depositions were visualized using a Stellaris 5 confocal system equipped with an inverted DMi8 microscope (Leica Microsystems, Wetzlar, Germany). Quantification of fluorescence intensity as corrected total cell fluorescence (CTCF) was performed using ImageJ software, version 1.53t. The data were normalized to the glucose-supplemented vehicle and represented in arbitrary units (a.u.).

### 4.10. Mitochondrial Mass and Potential Assay

The mitochondrial membrane potential (_Δ_Ψm) was assessed using the TMRE dye, and mitochondrial mass was evaluated with the MTG dye. The co-staining procedure followed previously established protocols [5,57]. Both dyes were diluted in heat-inactivated, tenfold-concentrated *E. coli* OP50 containing the *cis*- or *trans*-harpagoside treatments to final concentrations of 100 nM for TMRE and 4 μM for MTG. For each experimental group, at least 1000 synchronized L4 worms were incubated at 20 °C overnight. Following staining, worms from each group were transferred to treatment and stain-free plates for 1 h to clear residual dye from the gut. Imaging was performed using a Stellaris 5 confocal system coupled with an inverted DMi8 microscope (Leica, Wetzlar, Germany). For each experiment, around 40 nematodes were randomly captured and in a blind manner at least 20 were scored for quantification of fluorescence intensity. Quantification was performed using ImageJ (version 1.53t), as previously described [5]. The experiment was performed in at least three independent biological replicates.

### 4.11. Detection of GFP-Fluorescence of SJ4143, CL2166, and LD1 Strains

For fluorescence detection in the transgenic *C. elegans* strains, worms were exposed to *cis*- and *trans*-harpagoside or Vehicle (+G) for 24 h, mounted on microscopy slides, and visualized. A brief heat stress (37 °C for 5 min) was applied as a positive control. Imaging was performed at 10× or 20× magnification using the GFP filter on a Leica Stellaris 5 confocal system equipped with an inverted DMi8 microscope. Fluorescence intensity was quantified using ImageJ software (version 1.53t) [5,57]. Quantification was normalized to the Vehicle (+G) group, and results were expressed as normalized CTCF.

### 4.12. Statistical Analysis

Statistical analyses were performed using SigmaPlot version 11.0 (Systat Software GmbH, Erkrath, Germany). Data are presented as mean ± standard error of the mean (SEM). Statistical significance was considered at *p* * < 0.05. When the assumption of normality was violated, non-parametric tests were applied (ANOVA on ranks followed by Dunn’s multiple comparison test). The quantitative HPLC results are presented as the mean assay ± relative standard deviation (% RSD, *n* = 3) from three sequentially injected aliquots of a single sample.

## 5. Conclusions

This study demonstrates that the previously unexplored *Scrophularia aestivalis* exhibits significant therapeutic potential, as several of its primary fractions showed significant anti-obesity properties in a glucose-supplemented *C. elegans* model. Our findings indicate that the secondary metabolites present, particularly *cis*- and *trans*-harpagoside, not only modulate lipid metabolism but also enhance mitochondrial function and activate SKN-1/*gst-4* pathway. Together, these results highlight *S. aestivalis* as a promising source of natural compounds for the development of plant-based anti-obesity therapies.

## Figures and Tables

**Table 2:** Quantity assessment of the *S. aestivalis* total methanolic extract and its primary fractions, presented as mg per g dry extract (μg/mgd. extr.).

Iridoid Glycosides (mg/g d. Extr. ± RSD)	SCA	SCA-1D	SCA-1E	SCA-1F	SCA-1G
*cis*-Harpagoside (**17**)	85.35 ± 0.42	58.25 ± 0.53	-	-	-
*trans*-Harpagoside (**19**)	45.17 ± 0.44	25.72 ± 0.28	501.06 ± 0.33	69.18 ± 0.48	121.45 ± 0.16
**total**	130.52	83.97	501.06	69.18	121.45

*d. extr.—dry extract; RSD—relative standard deviation*.

**Table 3:** Concentration of *cis*- and *trans*-harpagoside in SCA and fractions (1D-G), expressed in both μg/mL and μM, respectively.

Iridoid Glycosides Concentrations (μg/mL; μM)	SCA	SCA-1D	SCA-1E	SCA-1F	SCA-1G
*cis*-Harpagoside (**17**)	8.54; 17.26	5.83; 11.78	-	-	-
*trans*-Harpagoside (**19**)	4.52; 9.13	2.57; 5.20	50.11; 101.33	6.92; 13.99	12.15; 24.56
**total**	13.06; 26.39	8.40; 16.98	50.11; 101.33	6.92; 13.99	12.15; 24.56

## Data Availability

The original contributions presented in this study are included in the article. Further inquiries can be directed to the corresponding authors.

## Supplementary Materials

The following supporting information can be downloaded at https://www.mdpi.com/article/10.3390/molecules30214202/s1, Table S1: 1H NMR data for *cis*- and *trans*-harpagoside (400 MHz, in CD3OD, δ in ppm, J in Hz); Table S2: UPLC-HRMS/MS mass spectrometric data of identified compounds in *S. aestivalis* fractions; Figure S1: UPLC-HRMS base peak chromatogram of SCA-1D and SCA-1E; Figure S2: UPLC-HRMS/MS base peak chromatogram of SCA-1F and SCA-1G; Figure S3: Calibration curves of (A) *cis*-harpagoside and (B) *trans*-harpagoside estimated by HPLC at 275 nm; Figure S4: HPLC-UV chromatogram of a standard solution containing: (1) *cis*-harpagoside (50 µg/mL) and (2) *trans*-harpagoside (110 µg/mL) at 275 nm.

## Abbreviations

The following abbreviations are used in this manuscript:

CTCF	Correlated total cell fluorescence
*gst-4*	Glutathione S-transferase 4
NRF2	Nuclear factor erythroid 2-related factor 2
PPAR-γ	Peroxisome proliferator-activated receptor gamma
SCA	*Scrophularia aestivalis*
SKN-1	Protein skinhead-1
TNF-α	Tumor necrosis factor-α
UPLC-HRMS/MS	ultra-high liquid chromatography with high-resolution mass spectrometry
