# Supplementary material for: Phytochemical Profiling and Anti-Obesogenic Potential of Scrophularia aestivalis Griseb. (Scrophulariaceae)
Source: Molecules. 2025 Oct 27;30(21):4202. doi: 10.3390/molecules30214202 (PMC12609081; doi:10.3390/molecules30214202)
Supplement: Supplementary file 1 [file molecules-30-04202-s001.zip › R1 Supplementary material.pdf]

Supplementary information

Phytochemical profiling and anti-obesogenic potential of *Scrophularia aestivalis* Griseb. (Scrophulariaceae)

Konstantina Priboyska[1], Monika N. Todorova[2], Vanya I. Gerasimova[1,3], Martina S. Savova[2,4], Slaveya Krustanova[1,3], Zhanina Petkova[1,3], Stoyan Stoyanov[5], Milena P. Popova[1,3], Milen I. Georgiev[2,4], Kalina Alipieva[1,3]

[1]Institute of Organic Chemistry with Centre of Phytochemistry, Bulgarian Academy of Sciences, 1113 Sofia, Bulgaria

[2]Laboratory of Metabolomics, Institute of Microbiology, Bulgarian Academy of Sciences, 4000 Plovdiv, Bulgaria

[3]Centre of Competence "Sustainable Utilization of Bio-resources and Waste of Medicinal and Aromatic Plants for Innovative Bioactive Products" (BIORESOURCES BG), Sofia, Bulgaria

[4]Center of Plant Systems Biology and Biotechnology, 4000 Plovdiv, Bulgaria

[5]Institute of Biodiversity and Ecosystem Research, Bulgarian Academy of Sciences, 1113 Sofia, Bulgaria

Table S1. ^{1}H NMR data for *cis*- and *trans*-harpagoside (400 MHz, in CD_3OD, δ in ppm, J in Hz).

Position	***cis*-harpagoside**	***trans*-harpagoside**
	δ_H (J)	δ_H (J)
Aglycone		
1	6.08 *d* (1.2)	6.18 *d* (1.2)
2	-	-
3	6.38 *d* (6.4)	6.41 *d* (6.4)
4	4.92 *dd* (6.4, 1.6)	4.94 *dd* (6.4, 1.6)
5	-	-
6	3.71 *dd* (4.5, 1.6)	3.77 *dd* (4.4, 1.4)
7	2.14 *brd* (15.1)	2.22 *brd* (15.1)
	1.96 *dd* (15.1, 4.5)	2.02 *dd* (15.1, 4.4)
8	-	-
9	2.90 *brs*	2.94 *brs*
10	1.49 *s*	1.54 *s*
Glucosyl		
1’	4.58 *d* (7.9)	4.63 *d* (7.9)
2’	3.21 *dd* (7.9, 9.1)	3.23 *dd* (7.9, 9.1)
3’	3.39 *t* (9.1)	3.41 *t* (9.1)
4’	3.34 *m*	3.35 *m*
5’	3.34 *m*	3.35 *m*
6’	3.84 *dd* (12.1, 1.8)	3.94 *dd* (12.1, 2.0)
	3.62 *dd* (12.1, 5.5)	3.73 *dd* (12.1, 5.7)
Cinnamoyl		
2’’, 6’’	7.55 *m*	7.59 *m*
3’’, 4’’, 5’’	7.35 *m*	7.40 *m*
α	6.95 *d* (12.6)	7.67 *d* (16.1)
β	6.00 *d* (12.6)	6.51 *d* (16.1)

Table S2. UPLC-HRMS/MS mass spectrometric data of identified compounds in *S. aestivalis* fractions.

№[a]	Rt[b], min	MF[c]	Exp. m/z [M-H]-, [M+HCOO]-	Calculated mass	Δ Mass, ppm	MS/MS product ions [m/z]	Identification	MS peak area			
								SCA-1D	SCA-1E	SCA-1F	SCA-1G
**1**	1.23	$C_{15}H_{22}O_9$	391.1246	346.1246	1.78	183.0656, 165.0549, 139.0549, 119.0549, 89.0229	*Aucubin*	2.027 E8	2.850 E7	Tr.	Tr.
**2**	1.63	$C_{15}H_{24}O_{10}$	409.1351	364.1369	0.76	201.0764, 183.0657, 165.0554, 179.0556, 119.0339	*Harpagide*	5.362 E8	1.209 E8	4.801 E7	2.528 E7
**3**	2.33	$C_{16}H_{24}O_{10}$	421.1359	376.1369	1.70	183.0663, 213.0769, 195.0657, 163.0395, 113.0239	*6-O-Methyl catalpol*	2.448 E8	4.923 E7	4.828 E6	Tr.
**6**	6.17	$C_{17}H_{26}O_{11}$	451.1462	406.1475	1.37	301.6126, 183.0653, 165.0549, 113.0233	*8-O-Acetylharpagide*	2.979 E8	5.135 E7	1.972 E7	5.154 E7
**8**	10.41	$C_{30}H_{38}O_{16}$	653.2102	654.2160	-3.54	377.1251, 325.8606, 315.1096, 309.0977, 291.0883, 187.0394, 181.0498, 163.0393, 145.0286, 119.0492	*Saccatoside*	Tr.	3.901 E9	1.660 E9	1.517 E9
**10**	11.07	$C_{30}H_{38}O_{16}$	653.2101	654.2160	-4.36	377.1250, 325.8607, 315.1096, 309.0977, 291.0883, 187.0394, 181.0498, 163.0394, 145.0286, 119.0492	*Premnacorymboside B*	Tr.	2.831 E9	1.785 E9	8.665 E8
**11**	13.61	$C_{30}H_{38}O_{16}$	653.2100	654.2160	-3.89	377.1250, 325.8607, 315.1096, 309.0977, 291.0883, 187.0394, 181.0498, 163.0394, 145.0286, 119.0492	*p-Coumaroyl rhamnopyranosylcatalpol isomer*	Tr.	1.784 E9	4.035 E8	2.484 E8
**a**	17.88	$C_{30}H_{38}O_{15}$	683.2205	638.2211	-1.46	361.1311, 215.0710, 163.0393, 147.0442	*Cinnamoyl rhamnopyranosylcatalpol isomer*	2.873 E8	5.251 E8	4.279 E8	4.694 E8
**b**	18.46	$C_{30}H_{38}O_{15}$	683.2205	638.2211	-0.58	361.1296, 215.0712, 163.0393, 147.0442, 113.0233	*Cinnamoyl rhamnopyranosylcatalpol isomer*	Tr.	Tr.	2.500 E9	1.833 E9
**13**	20.03	$C_{30}H_{38}O_{15}$	683.2205	638.2211	-1.89	361.1303, 281.0814, 215.0710, 163.0392, 147.0442, 113.0233	*6-O-α-L-(2"-O-trans-Cinnamoyl)rhamnopyranosylcatalpol*	Tr.	Tr.	1.609 E8	1.240 E8
**15**	20.50	$C_{24}H_{30}O_{12}$	509.1671	510.1737	1.25	201.0764, 183.0654, 163.0392, 145.0285, 119.0491	*p-coumaroyl harpagide* [24]	Tr.	2.980 E8	2.920 E8	1.219 E8
**16**	20.72	$C_{30}H_{38}O_{15}$	683.2206	638.2211	-1.93	361.1297, 311.0944, 215.0709, 163.0393, 147.0441, 113.0239	*6-O-α-L-(3"-O-trans-Cinnamoyl)rhamnopyranosylcatalpol*	Tr.	Tr.	2.396 E8	1.240 E8
**c**	24.22	$C_{31}H_{40}O_{15}$	651.2308	652.2367	-1.96	475.1857, 329.1242, 193.0502, 175.0394, 113.0233	*Martynoside* [13,24]	Tr.	Tr.	6.404 E8	1.065 E8
**17**	24.62	$C_{24}H_{30}O_{11}$	539.1775	494.1788	0.88	183.0658, 165.0549, 147.0442, 103.0540	*cis-Harpagoside (8-O-(Z)-Cinnamoylharpagide)*	7.413 E9	7.821 E8	2.024 E8	2.360 E8

**18**	25.31	$C_{28}H_{32}O_{14}$	637.1788	592.1792	1.26	283.0616, 162.446	*Linarin (Acacetin-7-O-rutinoside)*	Tr.	Tr.	Tr.	3.466 E7
**19**	27.45	$C_{24}H_{30}O_{11}$	539.1778	494.1788	-1.45	207.0663, 183.0656, 165.0580, 147.0443, 139.0391, 113.0233	*trans-Harpagoside (8-O-(E)-Cinnamoylharpagide)*	6.846 E8	1.078 E10	4.117 E9	3.703 E9

[a]Numbers of the assigned compounds corresponds to the elution order, compounds a-c were detected only in the studied fractions; [b]Rt – retention time; [c]MF – molecular formula; Tr. – traces

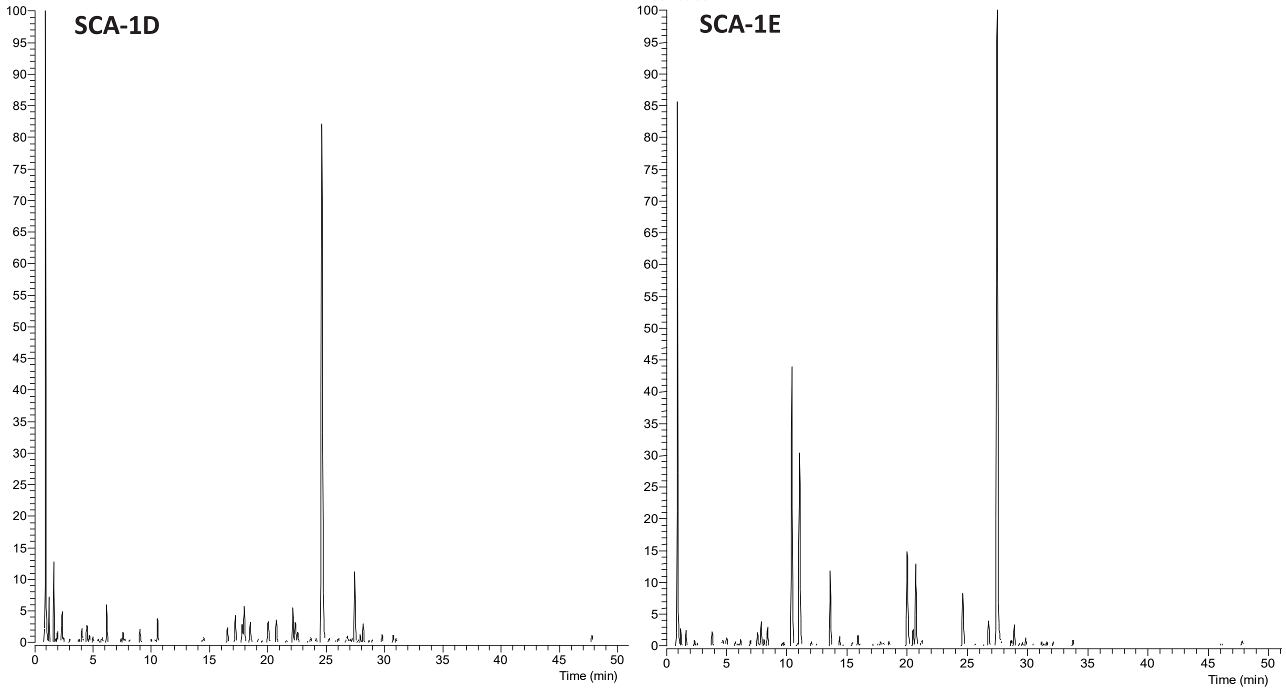

Figure S1. UPLC-HRMS base peak chromatogram of SCA-1D and SCA-1E.

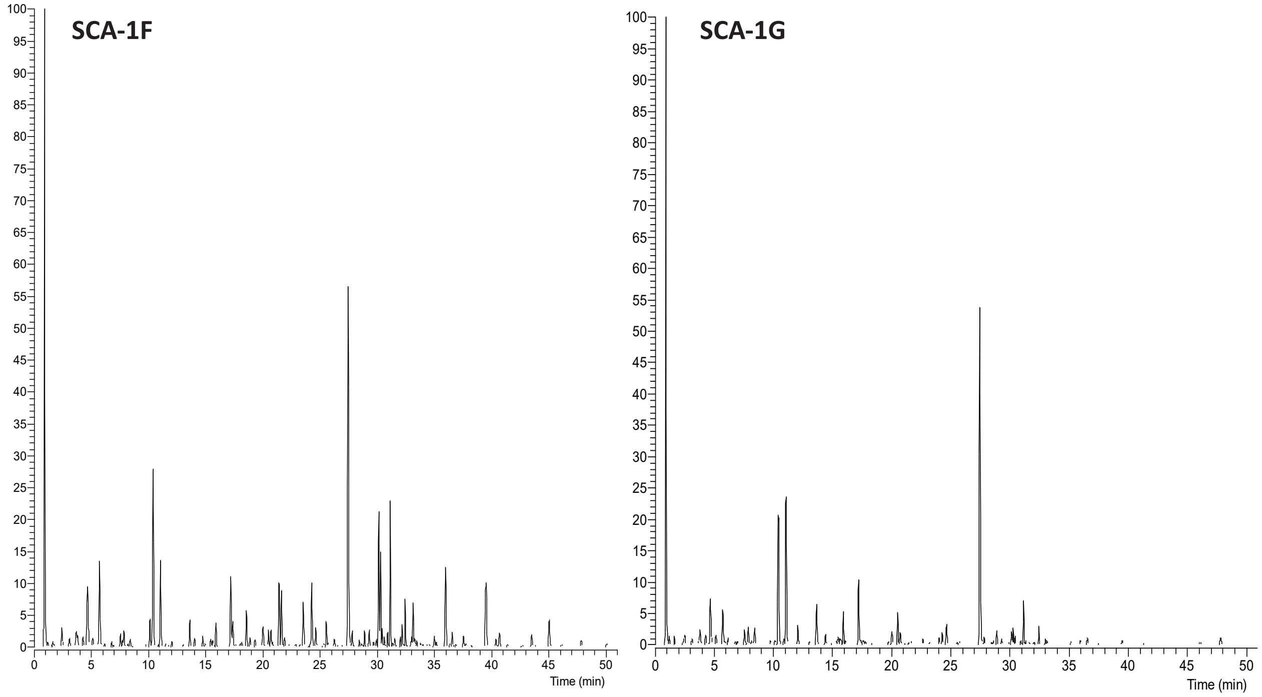

Figure S2. UPLC-HRMS/MS base peak chromatogram of SCA-1F and SCA-1G.

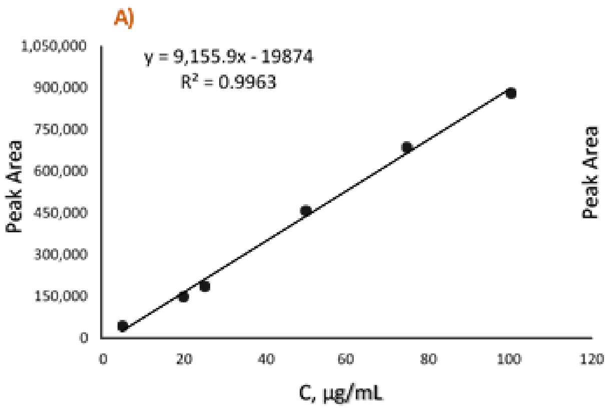

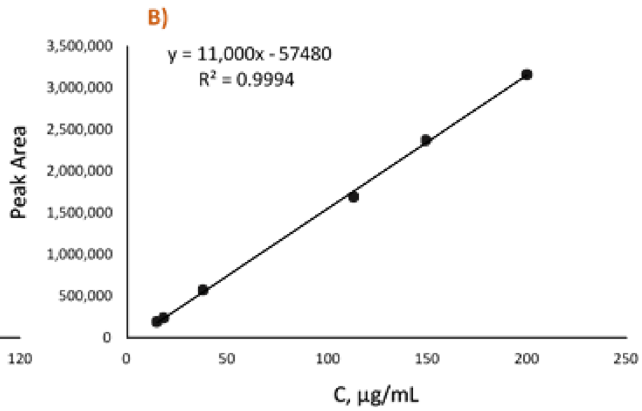

Figure S3. Calibration curves of A) *cis*-harpagoside and B) *trans*-harpagoside estimated by HPLC at 275 nm.

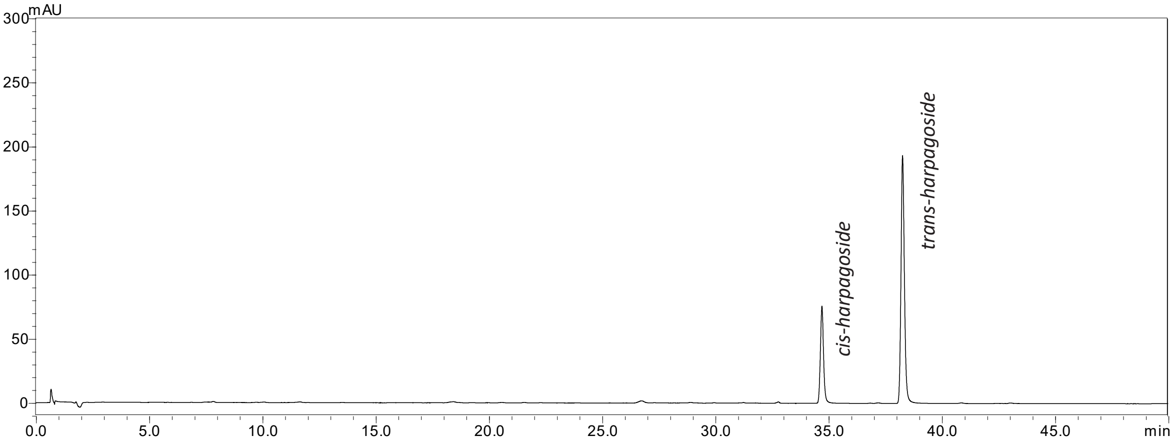

Figure S4. HPLC-UV chromatogram of a standard solution containing: 1) *cis*-harpagoside (50 µg/mL) and 2) *trans*-harpagoside (110 µg/mL) at 275 nm.
