# Supplementary material for: Phytochemical Profiling and Anti-Obesogenic Potential of Scrophularia aestivalis Griseb. (Scrophulariaceae)
Source: Molecules. 2025 Oct 27;30(21):4202. doi: 10.3390/molecules30214202 (PMC12609081; doi:10.3390/molecules30214202)
Supplement: Supplementary file 1 [file molecules-30-04202-s001.zip › trans harpagoside.zip!/Sca-5E/10/pdata/1/email_Sca-5E_10_1.pdf]

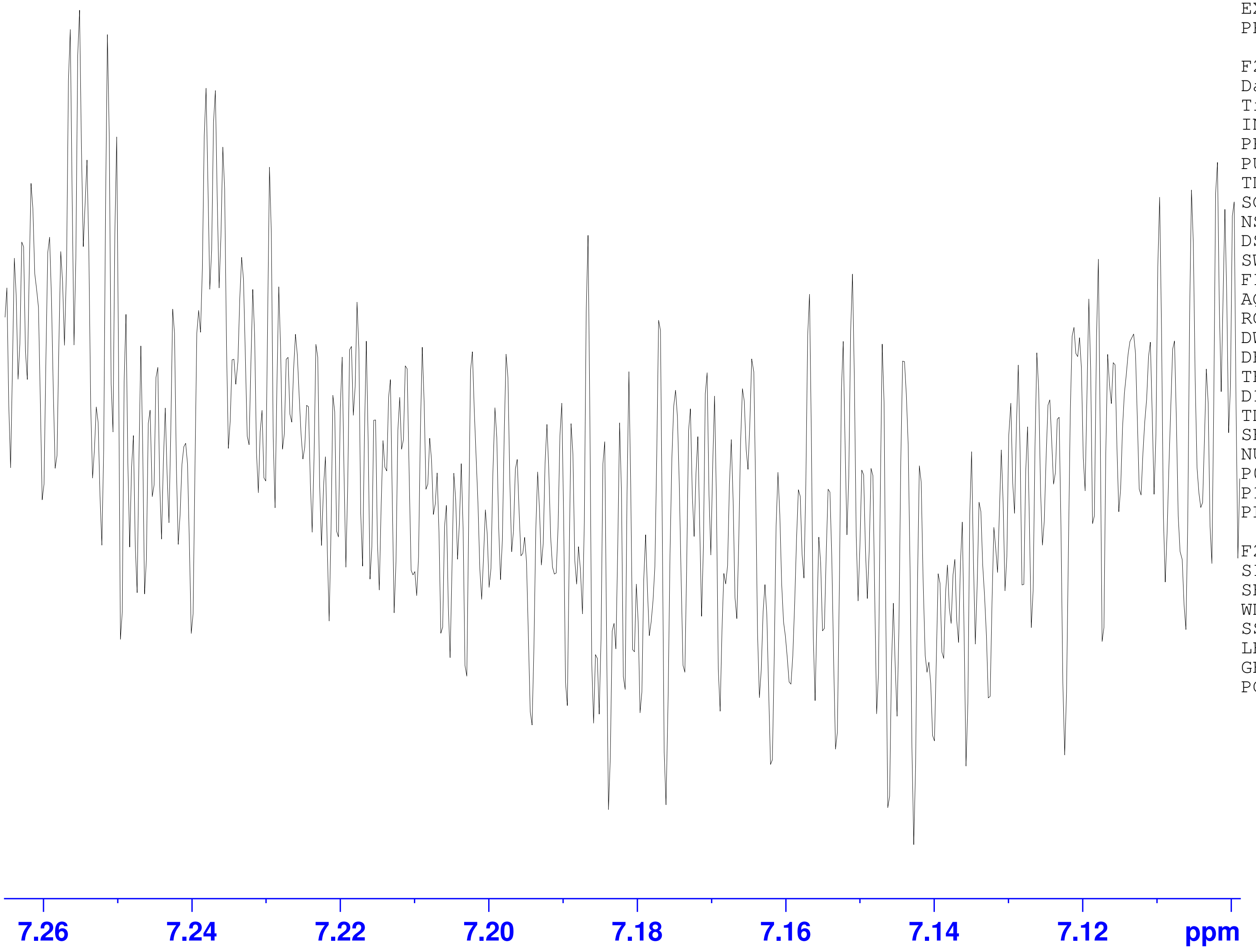

Current Data Parameters

NAME	Sca-5E
EXPNO	10
PROCNO	1

F2 - Acquisition Parameters

Date_	20240306
Time	0.22 h
INSTRUM	Avance Neo 400
PROBHD	Z175272_0007 (
PULPROG	zg30
TD	32768
SOLVENT	MeOD
NS	1
DS	0
SWH	6578.947 Hz
FIDRES	0.401547 Hz
AQ	2.4903679 sec
RG	101
DW	76.000 usec
DE	7.79 usec
TE	298.0 K
D1	2.00000000 sec
TD0	1
SFO1	400.1330415 MHz
NUC1	1H
P0	3.33 usec
P1	10.00 usec
PLW1	20.07200050 W

F2 - Processing parameters

SI	65536
SF	400.1300479 MHz
WDW	EM
SSB	0
LB	0 Hz
GB	0
PC	1.00
