# Supplementary material for: Phytochemical Profiling and Anti-Obesogenic Potential of Scrophularia aestivalis Griseb. (Scrophulariaceae)
Source: Molecules. 2025 Oct 27;30(21):4202. doi: 10.3390/molecules30214202 (PMC12609081; doi:10.3390/molecules30214202)
Supplement: Supplementary file 1 [file molecules-30-04202-s001.zip › cis harpagoside.zip!/Sca-8A/10/pdata/1/email_Sca-8A_10_1.pdf]

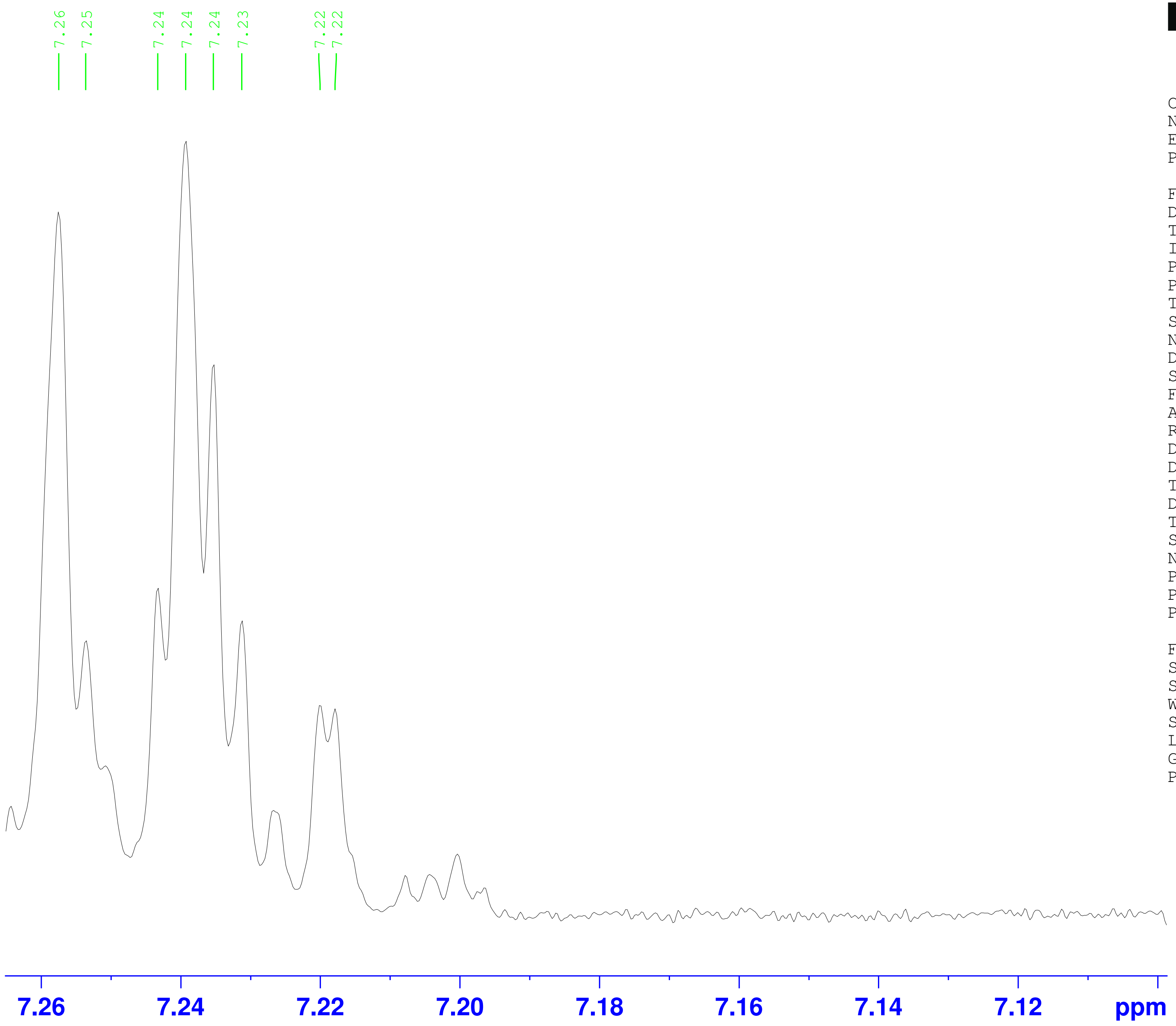

Current Data Parameters
NAME Sca-8A
EXPNO 10
PROCNO 1

F2 - Acquisition Parameters
Date_ 20240318
Time 17.15 h
INSTRUM Avance Neo 400
PROBHD Z175272_0007 (
PULPROG zg30
TD 32768
SOLVENT MeOD
NS 1
DS 0
SWH 6578.947 Hz
FIDRES 0.401547 Hz
AQ 2.4903679 sec
RG 101
DW 76.000 usec
DE 7.79 usec
TE 298.0 K
D1 2.00000000 sec
TD0 1
SFO1 400.1330415 MHz
NUC1 1H
P0 3.33 usec
P1 10.00 usec
PLW1 20.07200050 W

F2 - Processing parameters
SI 65536
SF 400.1300477 MHz
WDW EM
SSB 0
LB 0 Hz
GB 0
PC 1.00
